# Turn-over rate of academic faculty at the College of Health Sciences, Addis Ababa University: a 20-year analysis (1991 to 2011)

**DOI:** 10.1186/1478-4491-11-61

**Published:** 2013-12-01

**Authors:** Alemayehu Hailu, Damen Haile Mariam, Daniel Fekade, Miliard Derbew, Amha Mekasha

**Affiliations:** 1 Addis Ababa University, School of Public Health, Addis Ababa, Ethiopia; 2 Addis Ababa University, School of Medicine, Addis Ababa, Ethiopia; 3 Medical Education Partnership Initiative (MEPI) Ethiopia, College of Health Sciences, AAU Addis Ababa, Ethiopia

**Keywords:** Faculty turnover rate, Ethiopia, Addis Ababa University

## Abstract

**Background:**

Faculty turn-over affects both workers and organizations. Turnover of faculty and researchers is increasing alarmingly and costing the universities and the country at large. Fast turnover of health professionals from the health system and from academic institutions has recently received substantial attention from both academia and health sector managers. This paper calculates the faculty turnover rate at the College of Health Sciences of Addis Ababa University during the period of September 1991 to August 2011.

**Methods:**

The study was conducted at the College of Health Sciences, Addis Ababa University. Retrospective analysis of employee records was done. All records of the faculty that were working in the College during the 20-year period, starting from September 1991 to August 2011 were retrospectively reviewed. Data were collected from the employee records accessed from the College’s human resources database and supplemented by payroll sheets and different reports. A structured checklist was used to extract the required data from the database. The crude turnover rate for academic faculty was calculated.

**Results:**

Within the 20-year period of September 1991 to August 2011, a total of 120 faculty members left. The overall turn-over rate was 92.8 %. The rate in the most recent five years (172 %) is 8.5 times higher than the rate for the first five years (20 %). The average retention period before the termination of an employment contract was 4.9 years. The top five departments where employment contracts were relatively higher include: Nursing 15 (15.6 %), Internal Medicine 12 (12.5%), Public Health 10 (10.4%), Pediatrics 9 (9.4%) and Surgery 9 (9.4%). About two thirds (66.6%) of the faculty who were leaving were at the ranks of assistant professorship and above.

**Conclusion:**

This study revealed that outflow of faculty has been continuously increasing in the period reviewed. This implies that the College had been losing highly skilled professionals with considerably higher costs in monetary terms. In this regard, an urgent response is required to retain or significantly decrease the outflow of faculty. Different motivation and retention mechanisms should be identified and implemented. Various modalities of faculty development programs should also be initiated.

## Background

Employees are not “owned” by organizations like any other asset and, as such, staff turnover is a reality for many organizations. It is natural and healthy for people to leave the organization from time to time as this allows for the introduction of fresh ideas and innovations, flexible career opportunities, and enhances satisfaction in the workplace [1]. On the other hand, unless organizations retain workers for a reasonable period, they are unlikely to be able to provide the quality services required to remain competitive [2].

The fate of a university depends on its ability to recruit and retain talented faculty members [2]. Faculty turnover is a pervasive feature of the employment market. Fast turnover of academicians affects both the faculties and the university. For faculty members that leave their employment, it can not be easy to learn new job-specific skills and find different career prospects. Universities will also lose job-specific skills, which will be disruptive to their teaching/learning, as well as to their service rendering processes [2,3]. Subsequently, fast turn-over of faculty increases the cost that the universities incur in their human resources development activities [4,5].

Currently, turnover of faculty and researchers is increasing alarmingly within the universities in Ethiopia. At the country level, the situation is aggravated by the high rate of brain drain. For example, in the 1960s and 1970s, faculty sent for study leave abroad came back immediately after completing their studies to return to their appointments. In recent times, however, the opposite is the case. A sizable number of Ethiopian academics have migrated abroad in search of better conditions [6-9].

Moreover, this fast turn-over of faculty as well as different professionals from academic institutions has not yet received due attention from both academics and health sector managers. Minimal, if any, effort has been given to investigate and understand the causes of such high turn-over in developing countries. Faculty turn-over is driven by certain identifiable characteristics such as types of workers, tasks, organizations and markets. Evidence elsewhere has revealed that it is possible to significantly reduce the occurrence of turn-over of clinicians and other faculty in schools of health and medical sciences. However, actions to prevent such occurrences are rarely seen [6,8,10].

The current analysis is aimed at investigating the magnitude and trend of turn-over of the faculty of the College of Health Sciences (CHS), Addis Ababa University (AAU). The result could also help to proactively act in and facilitate decisions in struggling to achieve quality medical education in Ethiopia.

## Methods

### Study design and data analysis

This study is a retrospective analysis of the official records of employees. There are a number of ways to measure the rate of employee turn-over. For this study, the crude turn-over rate (CTR) is used. Crude turn-over rate is calculated based on a formula that is the number of those leaving in a given period divided by the average number of faculty members during the same period multiplied by 100. The rate was calculated separately for each consecutive five years using the same formula. The numerator includes all people leaving, even people who left involuntarily due to dismissal, redundancy or retirement.

Frequency and percentage was calculated to characterize the study subjects based on their socio-demographics, academic rank and their respective departments. The faculty turn-over rate was calculated for each five years starting from September 1991 to August 2011. Length of stay was computed to identify the magnitude of difference in longevity within the College. Early leavers were those who stayed for less than four years; middle leavers were those who stayed for four to eight years, and later leavers were those with longevity of more than eight years.

### Study subjects and data sources

The study was conducted in the CHS, AAU. The CHS was established in 1964 as a Faculty of Medicine with the goal of producing medical doctors to handle the country’s health problems. The Faculty of Medicine is the oldest and the largest among the health training institutions in the country, staffed with the most senior specialists in the country.

A retrospective cohort of employed faculty working at the CHS was followed for 20 years starting from September 1991 to August 2011 (*Meskerem* 1984 to *Pagume* 2003 in the Ethiopian Calendar). The term ‘faculty’ means the staff members with academic positions of lecturer and above academic rank. Data were collected from the records of the employees accessed from the College’s human resources database, payroll sheets and other relevant reports. The checklist was prepared to extract the required data from the database. Responsible people in the college were informed by a formal letter about this study and the required data. Any identification of the employee was not recorded anywhere on the checklist and appropriate measures were also taken to ensure confidentiality of information.

## Results

### Currently working faculty

Table 1 displays the distribution of currently working male and female faculty across different departments within the College. Out of a total of 253 faculty members in the College, the vast majority (85.4%) were males. More than 50% of the total faculty members were employed by 5 of the 18 departments within the College: Departments of Surgery 42 (16.2%), Internal Medicine 34 (13.4%), School of Nursing 25 (9.9%), Public Health 21 (9.1%) and Pediatrics and Child Health 16 (6.3%). Departments of Obstetrics/Gynecology, Microbiology and Psychiatry each had 12 (4.7%) academic faculty members.

**Table 1:** Distribution of faculties by sex and their departments, September 2011, Addis Ababa

		**Academic rank**				**Total**	
		**Profs.**	**Asso. Profs**	**Assi. Profs**	**Lec. s**	**N**	**%**
***Sex***	Female	1	4	18	14	37	14.6
	Male	17	38	127	34	216	85.4
***Department***	Surgery	2	5	29	5	41	16.2
	Internal medicine	1	4	24	5	34	13.4
	Nursing	0	0	4	21	25	9.9
	Public health	3	4	13	3	23	9.1
	Pediatrics	1	2	13	0	16	6.3
	GYN-OBS	1	2	9	0	12	4.7
	DMIP	4	3	1	4	12	4.7
	Psychiatry	0	6	6	0	12	4.7
	Radiology	0	3	7	0	10	4.0
	Ophthalmology	0	3	7	0	10	4.0
	Physiology	3	1	3	2	9	3.6
	Dermatology	0	1	7	0	8	3.2
	Pharmacology	1	0	3	4	8	3.2
	Anatomy	0	3	1	3	7	2.8
	Biochemistry	1	1	4	1	7	2.8
	Orthopedics	0	2	5	0	7	2.8
	Anesthesiology	0	0	6	0	6	2.4
	Pathology	1	2	3	0	6	2.4
	Total *N*	18	42	145	48	253	100
	%	7.1	16.6	57.3	19.0	100	

In terms of academic rank, more than half (57.3%) of the faculty members were assistant professors, one sixth (16.6%) were associate professors, 7.1% were professors, while the remaining 19% were lecturers. Out of a total of 18 professors in the College, only one is a female.

The Department of Microbiology had the largest number of professors [4] compared with other departments in the College, followed by the Department of Physiology [3] and the School of Public Health [3]. Of the four major clinical departments, surgery had two professors while the rest had one each. Departments of Biochemistry, Pathology and Pharmacology had one professor each (Table 1).

### Faculty turnover

**Figure 1:**
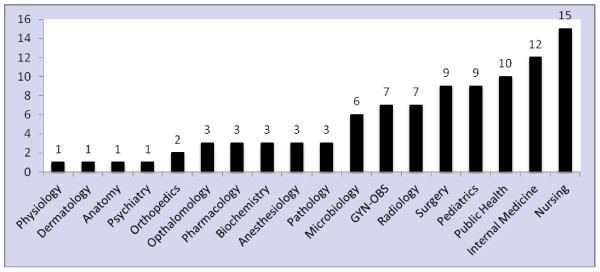
Frequency distribution of academic staff leaving from different departments (1991 to 2011).

Within the 20 year period (September 1991 to August 2011), a total of 120 faculty members left for various reasons. Out of these, data were obtained only for 96. The top five departments where higher numbers of employees left include: Nursing, 15 (15.6%), Internal Medicine, 12 (12.5%), Public Health, 10 (10.4%), Pediatrics, 9 (9.4%) and Surgery, 9 (9.4%). On the other hand, the Departments of Anatomy, Dermatology, Physiology and Psychiatry each lost only one faculty member within the specified period (Figure 1).

**Figure 2:**
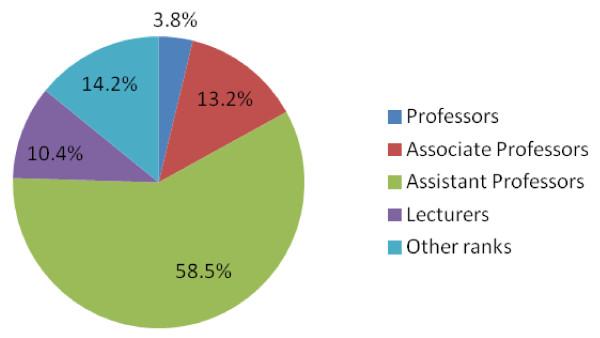
Distribution of leaving faculties across different academic ranks (1991 to 2011).

Out of the 120 faculty members who were leaving, data on academic rank were obtained for 106 of them. Accordingly, the last academic rank achieved before the termination of the employment contract was assistant professor for more than half (58.5%), while 13.2% of them were associate professors during the termination of their employment contracts (Figure 2).

Among leavers, the mean longevity at the College was 4.9 years (with a standard deviation of 3.8 years and a range of 1 month to 18 years) (Table 2).

**Table 2:** Sex and years of service of the staff members leaving (1991 to 2011)

	**N**	**%**
**Sex**		
Male	107	89.2
Female	13	10.8
**Service year (n = 80, 21 = missing)**		
>10	6	5.9
(9 to 10)	6	5.9
(7 to 8)	12	11.9
(5 to 6)	16	15.8
(3 to 4)	11	10.9
<3	29	28.7
Total	120	100

**Figure 3:**
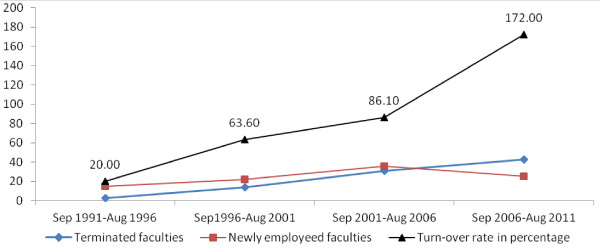
Staff turnover rate in the last 20 years: September 1991 to August 2011.

Among faculties employed during the overall period of analysis, a total of 120 faculty members were leaving due to different reasons, with an overall 20-year turn-over rate of 92.8%. The turn-over rate for the last five years (172%) was two times higher when compared with that of the preceding five years (Figure 3).

## Discussion

The College of Health Sciences at Addis Ababa University comprises the Schools of Medicine, Pharmacy, Public Health and Allied Health Sciences. The core functions of the College and the institutions under it include teaching, research and community service. According to the findings of the present study, the turn-over rate in the College was very high (92.8%), indicating that nearly all faculty members were replaced with new staff during the 20 years of analysis. The figure for the overall, last 20 years, turnover rates (92.8%) seems that the turnover is stable. A lot of faculty members had been assigned in the early two to five years of the study period due to the expansion of the university. But the reality is quite alarming in the most recent five years (172%). For developing countries like Ethiopia where human resources are limited, and in a situation where the number of senior faculty members is few, the depicted magnitude is quite alarming.

In terms of academic rank, the majority of the faculty members leaving were assistant professors. Most of the faculty members are medical doctors with specialty training in various fields, indicating that the College is losing highly skilled professionals with cost implications that are devastating. This shows the need for an urgent response to retain faculty or to significantly decrease their outflow.

Turn-over of faculty in the health and medical sciences is mostly due to a combination of attrition (through long-term illness and death, resignation, retirement, dismissal) and transfers (lateral, promotion, study leave). The main issue in most countries is the high rate of transfers of health professionals from public institutions to private and non-governmental organizations - seeking higher pay and incentives [6,8,10,11]. Departments with high turn-over may require more attention and positions with high turn-over may need to be restructured to be more interesting [11].

A high level of faculty turn-over could be caused by many other factors. Recruiting and seeking the wrong employees in the first place is the main factor causing fast turn-over in most organizations, and this may apply in the case of the CHS, AAU as well. Poor morale and a low level of motivation within the workforce, and a mismatch among the employee’s personal values, career, goals and plans with the larger corporate culture are also other factors for fast turn-over. A buoyant local labor market offering more attractive opportunities to employees also increases the rate.

Different studies indicate that salary, gender, age, position/title or academic rank, absences and average number of previous jobs as being significantly associated with whether employees remain within or leaving the organization. Study findings also revealed that higher employee turnover is also affecting the stability of the organization through eliminating skilled professionals from the university [3].

This study is only limited to measuring the faculty turnover rate using retrospective human resource data. Our study could not investigate the reasons why the faculty members were terminating their employment contract with the university due to the data limitation we faced. Detailed analysis of turn-over, motivating and demotivation factors, investigation of employee behavior to pinpoint why they leave the University and specifically their departments and what can be done to retain them are required. We also recommend that the employee record should be well organized, documented and digitalized so that it could serve for further analysis.

## Conclusion

As has been found in other studies, our findings revealed that retaining high caliber academic staff was a serious challenge to the college of Health Sciences at Addis Ababa University due to different reasons. The average retention period before the termination of the employment contract of faculty members was 4.9 years.

As this study revealed, the outflow of academic faculty is continuously increasing. Therefore, an urgent response is required to fully retain or balance the outflow, including the consideration of different motivations and retention mechanisms (such as salary increases and some other non-monetary incentives, promotion prospects, provision of job opportunities for spouses, a conducive and friendly atmosphere, overseas attendance at conferences, participating in the university’s community outreach and administrative services and academic promotional opportunities).

## Competing interests

The authors declare that they have no competing interests.

## Authors’ contributions

All authors (AH and DH, MD, DF and AM) participated in the conception of the research idea, design of the study, analysis of the data, interpretation, write-up and manuscript preparation equally. All the authors read and approved the final manuscript.
